# Synergizing Intelligence and Building a Smarter Future: Artificial Intelligence Meets Bioengineering

**DOI:** 10.3390/bioengineering10060691

**Published:** 2023-06-06

**Authors:** Daniele Giansanti

**Affiliations:** Centre Tisp, Istituto Superiore di Sanità, 00161 Rome, Italy; daniele.giansanti@iss.it; Tel.: +39-06-4990-2701

Smart Engineering (SE) describes the methods, processes, and IT tools for the interdisciplinary, system-oriented development of innovative, intelligent, networked products, production plants, and infrastructures [1]. These aspects are also positioned in the realm of “Industry 4.0”.

The exhaustive exchange of information among all parts of a smart system plays a key and major role. A smart system allows the improvement of client acceptance, quality, and cost saving.

The SE uses the following elements [2,3]:

A robust, stable, and performant network connection among the sub-systems; this can be either a wired or wire-less network.

The monitoring of the sub-systems’ running, by means of proper sensorized chains, with sensor devices.

The tuning of the sub-systems using proper activation chains, with actuator devices.

Innovative devices (e.g., robotics and miniaturized sensors).

Artificial Intelligence and/or IoT to assist with the interconnection and the decision processes.

The four pillars of data analytics (domain knowledge, math and statistics skills, computer science, communication, and visualization) integrated in the distributed system allow us to review data to identify the needed key insights and to solve problems.

SE is gradually establishing itself [4,5] as a technological approach capable of offering a wide range of highly innovative opportunities in the health domain.

The use of SE, individually (but also in hybrid and/or integrated systems) in robotics, wearable technology, portable devices, telemedicine, and biotechnology, is opening new horizons for improving the quality of care and people’s health.

On the one hand, increasing demand and challenges are emerging in this area [4,5]. On the other hand, there is also the need to prepare new professional figures able to face these challenges [6].

Care robots (CR)s are transforming the processes of care, therapy, assistance and rehabilitation [7]. The Policy Department for Economic, Scientific and Quality of Life Policies of the European Parliament categorized the (CR)s [8] into the following four groups:

- Robotic surgery.
- Care and socially assistive robots.
- Rehabilitation systems.
- Training for health and care workers.

Therefore, SEs can lead to important developments ranging from surgical robots to social robots, where important legal and ethical implications have to be addressed at the same time [9,10].

Wearable technology [11,12,13,14,15] and portable devices are revolutionizing citizen health monitoring. These sensors, also integrated with artificial intelligence (AI) [16] components, allow you to monitor, collect, and process information relating to biomedical parameters that can be used in a personalized way based on the patient’s health. Sensors built into clothing, watches, bracelets, and other devices allow you to collect real-time data.

Medical devices have benefitted from and will continue to benefit from SEs, whether they are hyper-miniaturized devices or macro-diagnostic devices. Thanks to the SE, both implantable and non-implantable devices can be remotely controlled and optimized even if this opens important questions about cybersecurity [17,18,19].

Telemedicine has a very long history. If used effectively and in accordance with regulations, it allows us to access care (including rehabilitation), monitoring and remote therapeutic treatment, without the need for physical meetings. Telemedicine has recently been applied in smart engineering [20,21,22] following the boost provided by the COVID-19 pandemic in this area, in addition to other areas [20].

Biotechnology is revolutionizing the field of personalized medicine. Gene therapies, regenerative medicine, and new discoveries in the field of molecular biology open new possibilities for the treatment of genetic, oncological, and degenerative diseases. The SE provides opportunities to facilitate the research, design, and production processes [23] of personalized drugs [24] and therapies, improving the efficacy and safety of treatments.

Collectively, the SE is transforming healthcare by offering innovative solutions for early diagnosis, personalized care, and remote healthcare [25]. These technologies promise to improve patients’ quality of life, reduce healthcare costs and open new frontiers in modern medicine.

The SE has been considered to be useful, in association with the specific technology, in the following macro areas of applications [25]: *assisting diagnosis and treatment, health management, disease prevention and risk monitoring, design and management of virtual assistants, design and management of smart hospitals, and assisting drug research (*Figure 1*)* [25].

From another perspective, it can be stated that the SE can play a strategic role in P7 MEDICINE (P7-M). P7-M is an interconnected medical management model based on molecular, cellular, and individual characteristics [26]. This model supports physicians, patients, laboratories, and pharmaceutical professionals to plan a personalized preventive and treatment plan for different health conditions. This interconnected model rests on the following “seven P”: *Personalized*, *Pervasive*, *Participatory*, *Predictive*, *Preventive*, *Programmable*, *and Perpetual* (P7), which are connected to healthcare. The SE is strategic, with the aim to design and manage complex models such as this one (Figure 2). New fields of research are oriented in this direction, investigating smart health, from a 360° technological point of view [27].

The growth of research in this area has been excellent in recent years. If we focus, for example, on two extremely different medical devices, i.e., wearable devices, and robotics, and analyze the growth of IoT and AI (two important components of smart engineering) in recent years, we are able to observe this growth.

In the case of wearable devices (*see Box 1 for the used composite keys*), based on a specific Pubmed search, 132 articles on IoT have been published since 2015, of which 102 (77.3%) have been published since 2020 after the outbreak of the COVID-19 pandemic. There are 205 articles on AI since 2008, of which 173 (84.5%!) have been published since 2020 after the outbreak of the COVID-19 pandemic.

Box 1 **The first proposed composite keys associated with wearable devices.**

*(artificial intelligence [Title/Abstract]) AND (wearable device)*

*(IoT [Title/Abstract]) AND (wearable device)*

In the case of robotics (*see Box 2 for the used composite keys*), based on a specific Pubmed search, 74 articles on IoT have been published since 2016, of which 66 (89.2%) have been published since 2020 after the outbreak of the COVID-19 pandemic. In addition, 681 articles on AI have been published since 1985, of which 493 (72.4%) have been published since 2020 after the outbreak of the COVID-19 pandemic.

Box 2 **The second proposed composite keys associated with robotics.**

*(artificial intelligence [Title/Abstract]) AND (Robot)*

*(IoT [Title/Abstract]) AND (robot)*

The information reported in this excursus highlights the interest in developing an initiative for discussion and scientific comparison in this area.

This Special Issue entitled “*Smart Engineering: Integrating Artificial Intelligence and Bioengineering*” [28], which acts as a forum of knowledge in this rapidly accelerating field addresses, both basic and applied research fields, while keeping in mind the ethical and regulatory issues.

The SE’s encounter with bioengineering has enormous potential in the health domain.

This has been found both in highly miniaturized devices (wearable devices), and in those with a high mechanical automation component (robotic devices), both in implantable and non-implantable medical devices, in telemedicine and biotechnology. The SE can cover the following applications: *assisting diagnosis and treatment, health management, disease prevention and risk monitoring, design and management of virtual assistants, design, and management of smart hospitals and assisting drug research*. It can also maintain and manage new healthcare models such as the *P7-M*. Targeted searches have shown the tremendous growth in the interest of scholars in this area, driven by their care in addressing this issue in a synergistic way through thematic editorial initiatives.

## Figures and Tables

**Figure 1:**
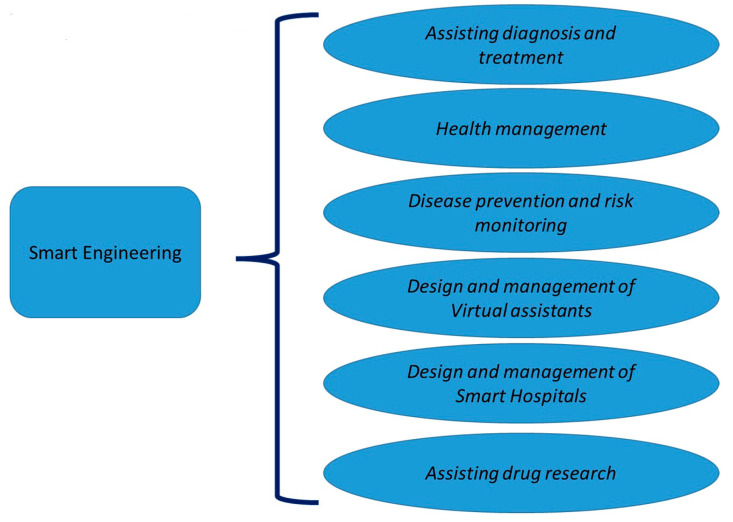
Applications of smart engineering in the health domain.

**Figure 2:**
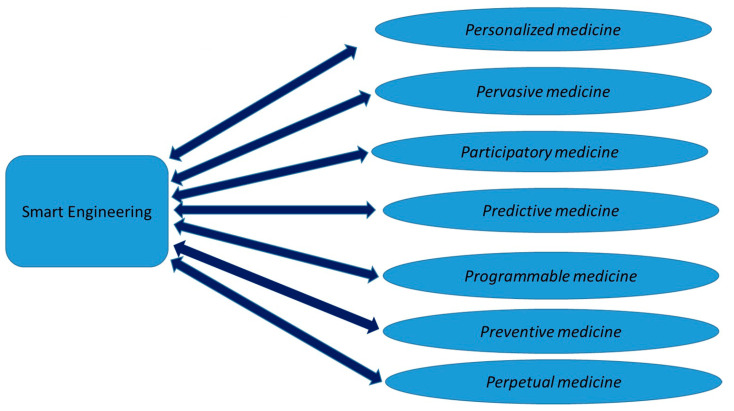
Smart Engineering and the 7-P medicine totally interconnected model.
